# Recombinant *Haemonchus contortus* 24 kDa excretory/secretory protein (rHcES-24) modulate the immune functions of goat PBMCs *in vitro*

**DOI:** 10.18632/oncotarget.13487

**Published:** 2016-11-21

**Authors:** Javaid Ali Gadahi, Baojie Li, Muhammad Ehsan, Shuai Wang, Zhenchao Zhang, Yujian Wang, Muhammad Waqqas Hasan, Ruofeng Yan, Xiaokai Song, Lixin Xu, Xiangrui Li

**Affiliations:** ^1^ College of Veterinary Medicine, Nanjing Agricultural University, Nanjing, PR China; ^2^ Department of Veterinary Parasitology, Sindh Agriculture University Tandojam, Pakistan

**Keywords:** H. contortus, ESP, 24 kDa, cytokines, proliferation, cell migration, Immunology and Microbiology Section, Immune response, Immunity

## Abstract

A 24 kDa protein is one of the important components in *Haemonchus contortus* (barber pole worm) excretory/secretory products (HcESPs), which was shown to have important antigenic function. However, little is known about the immunomodulatory effects of this proteinon host cell. In the present study gene encoding 24kDa excretory/secretory protein (HcES-24) was cloned. The recombinant protein of HcES-24 (rHcES-24) was expressed in a histidine-tagged fusion protein soluble form in *Escherichia coli*. Binding activity of rHcES-24 to goat PBMCs was confirmed by immunofluorescence assay (IFA) and its immunomudulatory effect on cytokine secretion, cell proliferation, cell migration and nitric oxide production were observed by co-incubation of rHcES-24. IFA results revealed that rHcES-24 could bind to the PBMCs. The interaction of rHcES-24 increased the production of IL4, IL10, IL17 and cell migration in dose dependent manner. However, rHcES-24 treatment significantly suppressed the production of IFNγ, proliferation of the PBMC and Nitric oxide (NO) production. Our findings showed that the rHcES-24 played important regulatory effects on the goat PBMCs.

## INTRODUCTION

 Haemonchosis is a disease of the small ruminant caused by a nematode parasite *Haemonchus contortus* (*H. contortus*)*;* it is most important and alarming challenges to the small ruminant's production. The infection of the *H. contortus* could cause high economic losses worldwide [1]. *H. contortus* is a blood feeding parasite and penetrates into the abomasal mucosa to feed the blood of the host and causing the anemia and decreased total plasma protein [2, 3].

*H. contortus e*xcretory/secretory (ES) molecules probably play important roles in the host-parasite interaction during infection process [4]. Previous studies revealed abundant expression of these molecules including sperm-coating protein (SCP)-like protein in the blood-feeding stages of *H. contortus* [5, 6]. These protein are found in a wide range of organism included arthropods, nematodes, flukes and plants [7-11] and considered prominent proteins in ES products [4]. In parasitic nematodes, *Ancylostoma*-secreted proteins (ASPs) were first described and reported that these proteins were abundant in the ES products of the infective stage (L3) assumed that, these proteins play a vital role in transition from free living to parasitic stage of hook worm [12, 13].

In *H. contortus*, two SCP proteins HcES-24 and Hc-40 have been recognized from the ES products [14-16]*.* HcES-24 was first identified as a low molecular weight antigen recognized by hyperimmune sera from the experimentally infected sheep with L3 and also same protein was identified from the ESPs of adult worm [17, 18]. In distinction to hookworms, ES-24 was identified in L4 and adult worms but not in eggs or L3s [15].

Previously we identified, that the *H. contortus* excretory and secretory products (HcESPs) displayed suppressive potential on the goat PBMCs *in vitro*. HcESPs inhibited the productions of IL-4, IFN-*γ*, increased the suppressive cytokine IL-10, enhanced the inflammatory modulator IL-17, suppressed the production of chemical factor NO, decreased the cell proliferation and activated the cell migration [19]. In our previous proteomic study of *H. contortus* excretory and secretory products (HcESPs) binding to goat PBMCs, SCP like protein was identified as a interacting protein to goat PBMCs at L4 to adult stages of *H. contortus in vivo* [20]. Binding of this protein to goat PBMCs at multiple stages *in vivo* indicated its role in the immune modulation. In the current study, the gene encoding 24 kDA (HcES-24) was cloned and the recombinant protein of HcES-24 (rHcES-24) was used to evaluate its regulatory effects on the goat PBMCs.

## RESULTS

### Cloning of HcES-24 gene

The amplicon of HcES-24 gene were successfully isolated by PCR of *H. contortus* cDNA with specific primers as designed above and a fragment of the correct size of 609 bp was obtained. The recovered PCR product was purified and successfully cloned into pMD19-T cloning vector which was confirmed by restriction enzyme digestion with *HindIII/Eco*RI restriction site enzymes.

### Construction and identification of the recombinant pET-32a (+)-HcES-24

The correct fragment of HcES-24 after sequencing was then inserted into *HindIII/Eco*RI sites of pET32a (+) vector. The recombinant plasmid was confirmed with restricted digestion . The digestion of recombinant pET32a-HcES-24 produced a fragment of about 609 bp which is equal to molecular mass of HcES-24. These results indicated that HcES-24 has been successfully inserted into pET32a vector.

### Sequence and phylogenetic analysis of HcES-24

**Figure 1:**
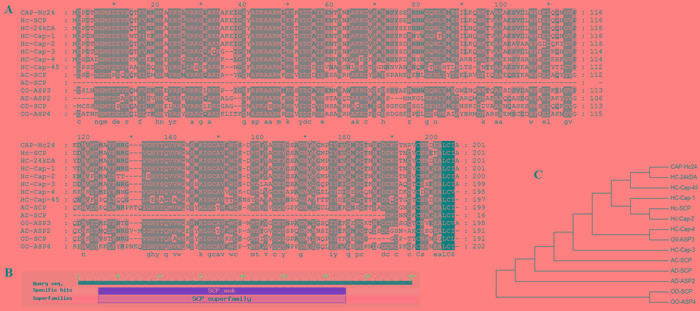
Multiple alignment of amino acid sequence of HcES-24 **A.** The amino acid sequence of HcES-24 aligned with CAP genes reported in the NCBI database HC-SCP: *H, contortus* *SCP extracellular domain containing protein CDJ92089.1 (98%),* HCES-24 kDA: *H, contortus* 24 kDa excretory/secretory protein AAC47714.1 (97%), HC-Cap-1: *H, contortus* cap-1 ALA23451.1 (95%), Hc-Cap-2: *H, contortus* cap-2 ALA23452.1 (94%), Hc-Cap-3: *H, contortus* cap-3 ALA23454.1 (93%), Hc-Cap-4: *H, contortus* cap-4 ALA23464.1 (78%), Hc-Cap-45: *H, contortus* cap-45 ALA23426.1 (69%), AC-SCP: *Ancylostoma ceylanicum* SCP-like protein EPB75408.1 (71%), AD-SCP: *Ancylostoma duodenale* SCP-like protein KIH59107.1 (6%), OO-ASP3: *Ostertagia ostertagi* C-type single domain activation associated secreted protein ASP3 CAO00416.1 (81%), AD-ASP: *Ancylostoma duodenale* secreted protein ASP-2 AAP41951.1 (64%), OD-SCP: *Oesophagostomum dentatum* SCP-like protein KHJ79965.1 (65%), OO-ASP4: *Ostertagia ostertagi* two-domain activation associated secreted protein ASP4 CAO00417.1 (67%). **B.** Putative conserved domain **C.** Phylogenetic tree of deduced amino acid sequences of HcCAP and other nematodes.

The isolated sequences were confirmed as HcES-24 gene by BLASTx, ORF contains 609 bp and encodes 202 amino acids. Multiple sequence alignment and Phylogenetic tree of the deduced protein sequence of HcES-24 with available sequences on NCBI database is shown in Figure 1. The multiple alignments of the deduced amino acid sequence indicated that HcES-24 was most closely related to *H. contortus SCP extracellular domain containing protein (98%), H. contortus* 24 kDa excretory/secretory protein (97%), *H. contortus* cap-2 (94%), *H. contortus* cap-3 (93%) and *H. contortus* cap-4 (78%).

### Expression and purification of rHcES-24

**Figure 2:**
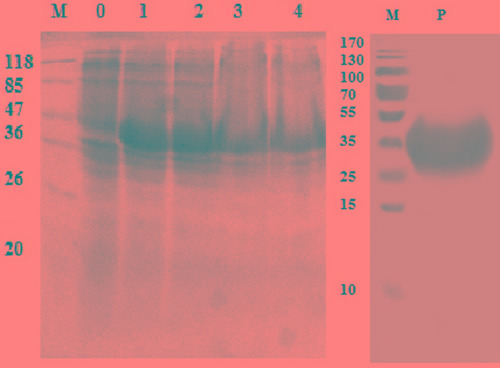
Expression and purification of rHcES-24 protein after induction with 1mM IPTG Lane M: standard protein molecular weight marker, 0: recombinant expression vector before induction, Lane 1-4 expression after induction at different time point and Lane P: purified rHcES-24 protein.

The rHcES-24 protein expressed in *E. coli* (BL21) cells as a double His 6 tagged fusion protein was purified. The expressed product was detected in SDS-PAGE after staining with coomassie brilliant blue (Figure 2). A protein band of rHcES-24 expressed product was about 42 kDa instead of the calculated molecular mass of ~24kDa due to extra pET-32a vector.

### Detection of rHcES-24 by western blotting

**Figure 3:**
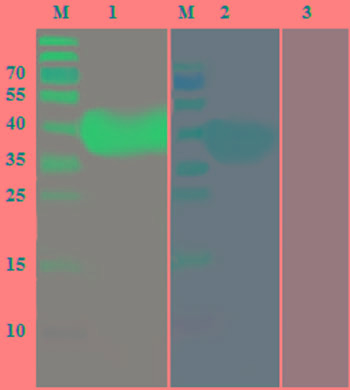
Western blot analysis of rHcES-24 Purified rHcES-24 were electrophoresed in SDS-PAGE **A.** and then transferred to a membrane for western blot analysis with rat anti- rHcES-24 sera **B.** and normal rat sera **C.** as control.

Western blot indicated that the recombinant protein was recognized by Rat anti rHcES-24, whereas, no protein was recognized by the normal rat serum (Figure 3).

### Binding of rHcES-24 to goat PBMCs

**Figure 4:**
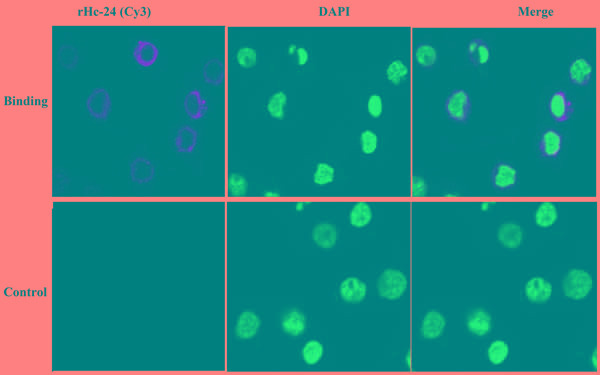
Binding of recombinant proteins rHcES-24 The nuclei of the corresponding cells were visualized by DAPI (blue) staining. Staining of the target proteins (red) were visualized by Cy3-conjugated secondary antibody. Merge, overlap of red and blue channels. No red fluorescence was observed in control group.

Binding of rHcES-24 to goat PBMCs was confirmed by immunofluorescence assay. Nuclei were stained with DAPI (blue fluorescence), and confocal microscopy images revealed that the rHcES-24 was bound to the cell surface (red fluorescence). In the control group, no red fluorescence was observed (Figure 4).

### Detection of the cytokine levels by ELISA

**Figure 5:**
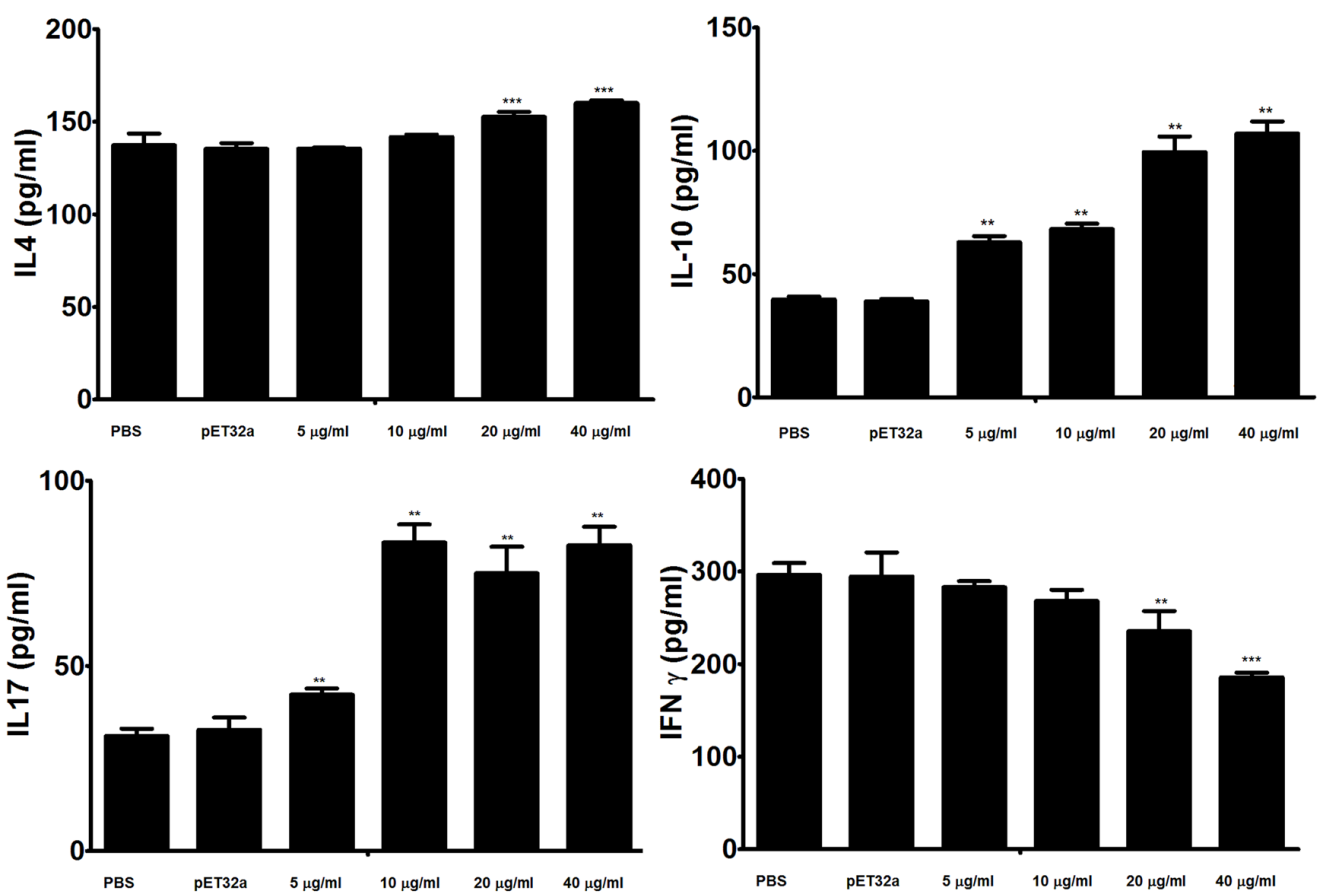
Analysis of the level of multiple cytokine production by PBMCs *in vitro* PBMCs were stimulated with ConA (10 μg/ml) for 24 h in the presence or absence of various concentrations of rHcES-24 and pET32a. Cytokine secretion in the supernatant of cell cultures was quantified by ELISA. The data are representative of three independent experiments (**p < 0.001, and ***p < 0.0001 *versus* the control).

Effects of the rHcES-24 on the cytokine production was analyzed by ELISA and results showed that rHcES-24 was significantly modulated the cytokine secretion in dose dependent manner (Figure 5). Production of type 2 cytokine IL-4 was significantly increased in PBMCs incubated with 20 and 40 μg/ml of rHcES-24. No significant difference was observed between the control and PBMCs treated with rHcES-24 at the dose of 5 and 10μg/ml. In the present study, secretion of IL-10 and IL-17 were also increased by the rHcES-24 in dose dependent manner. On the contrary, type II interferon (IFNγ) was suppressed by rHcES-24 in dose dependent manner.

### Cell migration assay

**Figure 6:**
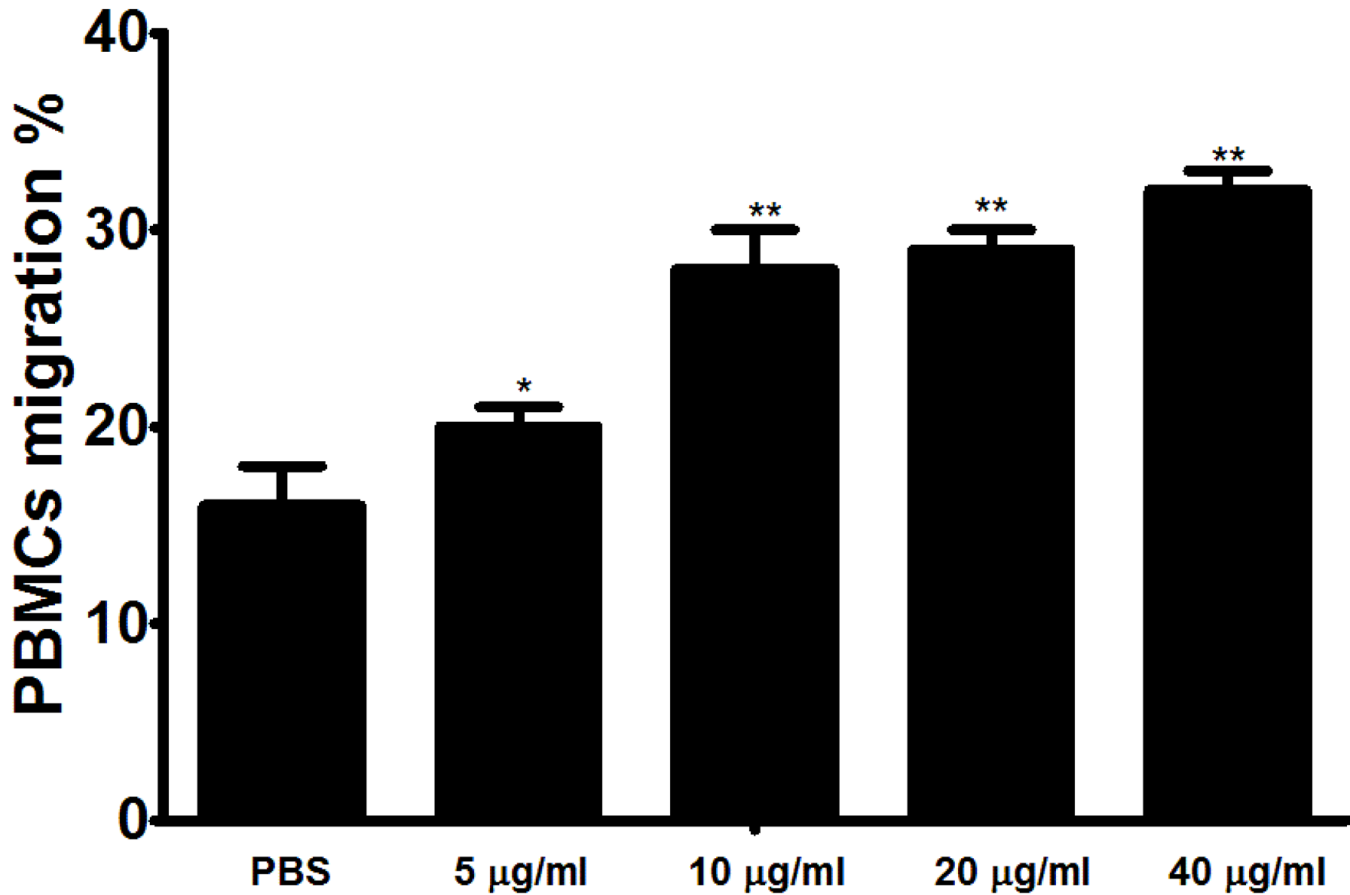
Impact of the various concentration of rHcES-24 on PBMC migration PBMC were treated with control buffer and different concentrations of rHcES-24. Then the random migration was determined. The difference between the mean values was calculated using ANOVA. Data are representative of 3 independent experiments; *p < 0.01 and **p < 0.001 *versus* the control.

To evaluate the impact of rHcES-24 on cell migration, cell migration assay was conducted (Figure 6). The percentage of migrated cells was significantly increased in cell treated with different concentration of rHcES-24.

### Cell proliferation assay

**Figure 7:**
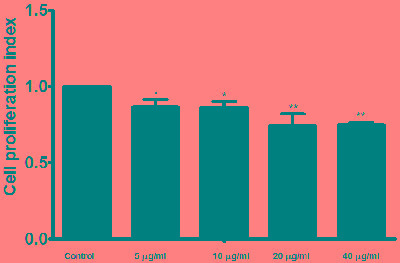
Effects of rHcES-24 on PBMCs proliferation Cells s were activated with ConA and incubated at the same time with serial concentrations of rHcES-24 at 37°C and 5% CO_2_. The proliferation was measured by CCK-8 incorporation after 72 h. Cell proliferation index was calculated considering the OD_450_ values in controls as 100%. The data were representative of three independent experiments (*p < 0.01 and **p < 0.001).

Cell counting kit (CCK8) was used to evaluate the effect of the rHcES-24 on the PBMC proliferation. rHcES-24 treatment significantly suppressed the proliferation of PBMC in dose dependent manner as compared with control group (Figure 7).

### Nitric oxide production assay

**Figure 8:**
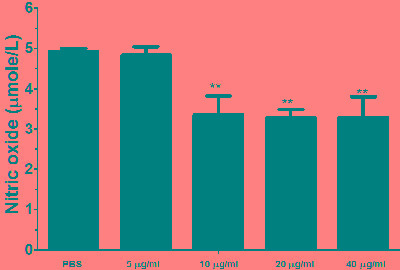
Effects of rHcES-24 on nitric oxide production by PBMCs *in vitro* Cells s were activated with ConA and incubated at the same time with serial concentrations of rHcES-24 at 37°C and 5% CO_2_. The nitrite concentration in the PBMCs was measured by using the Griess assay and used as an indicator of nitric oxide production by the PBMCs. The data were representative of three independent experiments (**p < 0.001).

Nitric oxide (NO) production by PBMCs treated with different concentration of rHcES-24 was measured by using the total nitric oxide assay kit. Results revealed that, rHcES-24 significantly suppressed the NO production by PBMCs (Figure 8).

## DISCUSSION

The helminth ESPs contain profuse amounts of CAP-superfamily proteins commonly referred to as activation-associated secreted proteins (ASPs) or sperm coating protein (SCP). ASPs are considered to play a key role in the switching from the free-living to the parasitic stage [21]. Determination of three dimensional protein structures of helminth ASPs suggested a vital role in a different regulatory function including altering the immune response [9, 22, 23]. Previously two proteins of CAP family Hc24 and Hc40 have been recognized from the ES products [14-16]. The role of *H. contortus* CAP protein particularly in immune regulation still not addressed. In the present study we cloned HcES-24 from adult *H. contortus* and rHcES-24 was used to evaluate the functional role in different regulations of goat PBMCs.

It is generally considered that the main protective immune responses against helminthes including *H. contortus* are Type 2 responses (Th2) associated with secretion of IL-4 and IL-5 [24-26]. In our recent study we found that HcESPs decreased the IL-4 production *in vitro* [19]*,* Contrary to that, in the present study, we identified that the rHcES-24 could enhance the secretion of IL-4. It indicated that this protein might be initiated the type 2 immune response against the infection of *H. contortus*.

The polarization of Th2 response during helminth infection is usually associated with T regulatory cells (T_Reg_). Inducible Treg (iTreg) cells are produced in the periphery and exert their suppressive activity mainly by producing IL-10, which are significantly linked with hyporesponsiveness and susceptibility to infection [27, 28]. IL-10 suppresses the development of allergic Th2 cell responses [29-31]. In our previous study we reported that, HcESPs increased the production of IL-10 in dose dependent manner [19]. Here we found that rHc-24 increased the production of IL-10. Hence, we can say that this protein play some roles in this stimulation of HcESPs on IL-10 production.

IL-17 a cytokine is produced by Th17 cells concerned in the regulation of inflammation [32]. IL-17 is associated with pathogenesis of various parasites including helminths [33-39]. Previously we reported that, HcESPs increased the production of IL-17 [19]. In the present study, we found that rHc-24 increased the production of IL-17; this indicated that rHc-24 could contribute some roles in the HcESPs on IL-17 production.

Cytokine gamma interferon (IFN-γ) secreted mainly from type 1 T cells (Th1) and NK cells also participates a key role in Th1 and Th2 differentiation [40]. Hsieh et al [41] reported that ESPs of *N. americanus* bind to NK cells and stimulated the Th1 responses by augmentation of IFN-γ production. Previously, it was reported that development of type1 responses produce the resistance to parasite and could inhibit the development of Th2 cells in the immune responses [42, 43]. Balance between Th1 and Th2 immune responses could determine the immunity and pathogenesis in parasitic infection [40]. Here, we found that PBMCs incubated with rHcES-24 suppressed the production of IFN-γ cytokine level in dose dependent manner. HcESPs suppress the production of IFN-γ [19], this protein also. Thus, we can say that this protein play putative role in this suppression of ESP on IFN-γ production.

Like other infections, parasites also induce immune cell trafficking to the site of infection, these cell can serve to control the pathogen [44]. Helminths may actively stimulate eosinophil and other lymphocyte migration to the sites of infection, resulting in tissue damage for the worm survival within the host [45, 46]. Previously reported that GIT nematodes produce a factors that stimulate the cell migration [47]. These finding indicated that helminthes could actively promote cell migration resulting in tissue damage and could provide favorable condition for their survival [46]. In our study rHcES-24 significantly increased the PBMC migration, Our findings fall within the our previous study about the effects of HcESPs on the PBMC migration *in vitro* [19] which indicated that, this secretory protein also one of the contributor of HcESPs that involved in the PBMCs migration.

An antigen-presenting cell (APC) and T cells can play important role in regulating the proliferation of immune cells and by this means alter the immune response [48] . Loke et al [48] reported that cell proliferative suppression induced by nematode *via* alternatively activated macrophages by IL-4. Ricci et al [49] demonstrated that hook worm crude antigen decreased the Treg cells and impaired cell proliferation. Previously we found that, HcESPs had a suppressive potential on the cell proliferation [19]. In current study, suppressive activity of the rHcES-24 on PBMC proliferation suggested that, HcES-24 protein is active molecule of HcESPs that impaired the cell proliferation.

Nitric oxide (NO) production considered to be mediating pro as well as anti-inflammatory [50]. NO has been associated with either a decrease in or aggravation of pathogenesis, increase in NO production help pathogen killing by host cell and may also increase the inflammatory responses [51, 52]. In previous studies reported, that NO could played considerable role in immunosuppression by its negative effects on lymphocyte proliferation [53]. [54-56]. Recently we reported that, HcESPs had negative effects on the NO production *in vitro* [19], therefore the suppression of the NO production by rHcES-24 indicated that HcES-24 might be played some roles in this suppression of HcESPs on NO production.

On the basis of our findings we concluded that HcES-24 is the very important and active protein of HcESPs that played crucial roles in the immune regulations. Our results demonstrated that IL-4, IL-10, IL17 and cell migration were increased by rHcES-24. However, cell proliferation and NO production by goat PBMCs were suppressed by rHcES-24. These findings will not only add to the understanding of the role of HcES-24, but might also help to illuminate the mechanisms involved in *H. contortus* immune evasion and host parasite interaction.

## MATERIALS AND METHODS

### Ethics statement

Animal experiments were conducted following the guidelines of the Animal Ethics Committee, Nanjing Agricultural University, China. All experimental protocols were approved by the Science and Technology Agency of Jiangsu Province. The approval ID is SYXK (SU) 2010-0005.

### Synthesis of *H. contortus* cDNA

Total RNA was isolated from Adult worm of *H. contortus* collected from the abomasums of donor goats as described previously [57]. The worms were ground using a pre-chilled mortor and pestle. One ml of Trizol (Invitrogen) was added and homogenized for 30 minutes. Then 200μl of Tri-chloromethane was added and the mixture was spun at 12,000rpm for 15 min at 4°C. After that, RNA was precipitated from the supernatant by the addition of 0.25 volumes of isopropyl alcohol per each milliliter of Trizol and incubated at -20°C for 30 min. The RNA was pelleted at 12,000 rpm at 4°C for 10 min. RNA pellet was washed by 70% ethanol, dried andresuspended in DEPC-treated water. RNA integrity was checked by agrose gel electrophoresis and quantified by NanoDrop ND-1000 Spectrophotometer. The RNA solution was used in subsequent cDNA preparation immediately. The cDNA was synthesized by reverse transcription reaction using cDNA Kit (TaKaRa Biotechnology) according to the manufacturer's instructions.

### Molecular cloning of HcES-24 and expression of recombinant HcES-24 protein (rHcES-24)

The complete open reading frame (ORF) of HcES-24 was amplified by reverse transcription-polymerase chain reaction (RT-PCR) using the designed primers of *H. contortus* 24kDa excretory/secretory protein mRNA, gene bank accession number AY821551.1.

The sense and antisense primer sequences are as the following: 5’-GAATTCATGTGTCCAGACACCAATG-3’ and 5’- AAGCTTTTATGGGGCAATACAGAGA -3’ which contained the initiation codon ATG with a *EcoRI* restriction site at the 5′-end and the terminus codon TAA with an *HindIII* restriction site at the 5 (underlined). PCR reaction and ampliﬁcation were performed using the Thermocycler PCR Machine (Biometra) under following condition: Initial denaturing at 94 ^o^C for 5 min (1 cycle), denaturing at 94 ^o^C for 1 min (35 cycles), annealing at 55 ^o^C for 1.5 min (35 cycles), extension at 72 ^o^C for 1.5 min (35 cycles) and final extension at 72 ^o^C for 10 min (1 cycle). The PCR products were purified by using E.Z.N.A. Gel Extraction Kit (Omega bio-tech, USA) and ligated into pMD19-T cloning vector (TaKaRa Biotechnology, China) and then transformed into *E. coli* DH_5α_ strain. The positive clones were confirmed by double digestion with *EcoRI/ HindIII* enzymes, and the selected positive recombinant clones were sequenced by Invitrogen Bio-tech (Shanghai, China). The sequence data was assembled and analyzed by DNAssist software version 2.2. The HcES-24 gene was then cloned into *EcoRI/ HindIII* sites of expression plasmid pET32a (+) vector (Novagen, USA). The recombinant plasmid was sequenced to confirm the correct insertion of HcES-24 gene in the proper reading frame.

The expression of the recombinant fusion protein in *E. coli* BL-2 1 cells (DE3) was induced by isopropy- ß D -thiogala ctoside (IPTG ) at a final concentration of 1mM for 4 h at 37°C in Luria-Bertini (LB) medium with ampicillin (100 μg/ml). The histidine-tagged fusion protein was purified from the supernatant of bacterial lysates using the His•Bind^®^Resin Chromatography kit (Novagen) and dialyzed in phosphate buffered saline (PBS, pH 7.4) to remove imidazole. Endotoxins were removed from the recombinant proteins using ToxinEraser^TM^ Endotoxin Removal kit (GeneScript, USA). The purity and concentration of the purified rHcES-24 was analyzed by 12% sodium dodecyl sulfate polyacrylamide gelelectrophoresis (SDS- PAGE ) followed by Coomassie blue staining.

### Sequence alignments and phylogenetic analysis of HcES-24

Sequence similarity was assessed using protein-protein basic local alignment search tools BLASTp and BLASTX sequences (http://www.blast.ncbi.nlm.nih.gov/Blast.cgi). HcES-24 sequences were aligned using ClustalX 1.83 program (http://www.clustal.org/). The phylogenetic tree was constructed by aligning the amino acid sequences using the Neighbor-Joining method and plotted and visualized using the Molecular Evolutionary Genetics Analysis 5.1 program (http://www.megasoftware.net/).

### Generation of polyclonal antibodies

To generate polyclonal antibodies against rHcES-24 , 0.4 mg of rHcES-24 was mixed with Freund's complete adjuvant (1:1) and injected subcutaneously into 3 female Sprague Dawley (SD) rats [58, 59]. Rats received four doses of injection with the same proteins at 2-week intervals. Ten days after the last injection, the rats were anesthetized with diethyl ether, and sera containing specific anti-rHcES-24 antibodies were collected. The concentration of antibodies was determined by ELISA. The specific reactivity with rHcES-24 was confirmed by western blot analysis.

### Immuno-blot for the rHcES-24

Purified rHcES-24 were resolved by 10% SDS-PAGE and transferred to polyvinylidene difluoride (PVDF) Membrane (Millipore, USA). Non-specific binding was blocked by incubating the membranes in 5% skim milk in Tris-buffered saline containing 0.1% Tween-20 (TBST) for 1 h at room temperature. The membranes were then washed 5 times (5 min each) with TBST, followed by incubation with the primary antibodies (anti-rHcES-24) for 1 h at 37 °C (1:100 dilution in TBST). After washing 5 times with TBST, the membranes were incubated with HRP-conjugated rabbit anti-rat IgG (Sigma, USA) for 1 h at 37 °C (diluted 1:2000 in TBST). Finally, the bound antibody was detected using 3, 3-diaminobenzidine tetra hydrochloride (DAB) kit (Boster Bio-technology) according to manufacturer's instructions.

### Binding of rHcES-24 to goat PBMC

Freshly isolated PBMCs were incubated in the presence and absence (control) of rHcES-24 (5μg/ml) for 1 h at 37°C. Confirmation of binding was determined by an immunofluorescence assay (IFA) as described by Yuan et al. [54]. Briefly, washed cells (10^5 ^/ ml) were fixed with 4% paraformaldehyde on a poly-L-lysine-coated glass slide. The cells were then treated with blocking solution (4% BSA in PBS) for 30 min to minimize background staining. After sequential incubation with rat anti-rHcES-24 IgG (1:100) for 2 h and a secondary antibody (1:300) coupled to the fluorescent dye Cy3 (Beyotime, Jiangsu, China) for 1 h, nuclear staining with 2-(4-amidinophenyl)-6-indole carbamidinedihydrochloride (DAPI, 1.5 μM; Sigma, MO, USA) was performed for 6 min. Then, protein localization was determined by observing the staining patterns with a 100× oil objective lens on a laser scanning confocal microscope (L SM710, Zeiss, Jena, Germany). Digital images were captured using the Zeiss microscope software package ZEN 2012 (Zeiss, Jena, Germany).

### Detection of the cytokine levels by ELISA of PBMCs treated with rHcES-24

The freshly isolated PBMCs were re-suspended to a final density of 5 × 10^6^ /ml in complete medium (RPMI 1640 supplemented with 100 U/ml penicillin, 100 μg/ml streptomycin, 2 mM L-glutamine, 10% FCS). In the test groups, cells were treated with ConA (10μg/ml) and different concentration of the rHcES-24 (5, 10, 20, and 40μg/ml). The control groups were treated with ConA in equal volume of PBS or ConA and recombinant protein of empty pET32a. Then, the cells were seeded into 24-well plates (1ml/well) and cultured for 24h in 5% CO2 atmosphere at 37 °C. The plates were then centrifuged at 200 × g for 15 min and the supernatants were collected. The levels of IL-4, IL-10, IL-17 and IFN-γ in supernatants were determined using commercially available goat ELISA kits (Jian cheng Biotech, China). The cell viability was assessed by means of the trypan blue exclusion test before the incubation of PBMCs with rHcES-24 . Three individual experiments were performed.

### Cell migration assay

The cell migration assay was performed using a Transwell system (Corning, USA), this allowed cells to migrate throughout an 8 μm pore size polycarbonate membrane [54]. The treatment group was incubated with different concentrations of rHcES-24 (5, 10, 20, and 40μg/ml) and the control group was treated with an equal volume of PBS. Cells that migrated through the membrane into the lower chamber were calculated with a Neubauer counting chamber and the results were presented as percentages of the seeded PBMC. Each experiment was performed in triplicate.

### Cell proliferation assay

Cell proliferation assay was performed as previously described [60]. Briefly, 100 μl of cell suspension (1 × 10^6^ cells/ml) were activated with ConA (10 μg/ml) and a serial concentrations of rHcES-24 (5, 10, 20, and 40μg/ml). The control group was treated with ConA in equal volume of PBS. The plate was cultured at 37°C and 5% CO_2_ for 72 h. Then 10 μl of CCK-8 solutions (Beyotime Biotechnology, China) were added to each well of the plates 4 h before harvesting and the absorbance values at 450 nm (OD_450_) were measured using a microplate reader (Thermo Scientific, USA). The OD_450_ of controls were set as 100%. Cell proliferation index was calculated by the formula: OD_450_ rHcES-24 /OD_450_ control. Each experiment was performed in triplicate

### Nitric oxide production assay

The goat PBMCs were harvested and washed twice with PBS. Then, 100 μl of cells (1 × 10^6^ cells/ml) were incubated either with PBS and a serial concentrations of rHcES-24 (5, 10, 20, and 40μg/ml) in 96-well plates in DMEM medium. Production of nitric oxide by PBMCs was determine d by measurement of intracellular nitrite in the PBMC by using the Griess assay [61] according to the instruction of Total Nitric Oxide Assay Kit (Beyotime Biotechnology, China). Absorbance of the colored solution at 540 nm (OD540) in each well was measured using a plate reader (Bio-Rad Laboratories, USA). Absorbance values were converted to micromoles per liter (μmol/L) using a standard curve that was generated by addition of 0 to 80 μmol/L sodium nitrite to fresh culture media. Three individual experiments were performed.
